# Microstructural and Mechanical Characterization of Ultra-Pure Aluminum for Low-Amplitude-Vibration Cryogenic Applications

**DOI:** 10.3390/ma19061195

**Published:** 2026-03-18

**Authors:** Mirko Pigato, Filippo Agresti, Alberto Benato, Carlo Bucci, Irene Calliari, Daniele Cortis, Serena D’Eramo, Shihong Fu, Cristina Giancarli, Luca Pezzato, Andrea Zambon, Antonio D’Addabbo

**Affiliations:** 1 Department of Industrial Engineering (DII), Università degli Studi di Padova, 35131 Padova, Italy; mirko.pigato@unipd.it (M.P.); irene.calliari@unipd.it (I.C.); a.zambon@unipd.it (A.Z.); 2 Institute of Condensed Matter Chemistry and Energy Technologies (ICMATE), National Research Council of Italy, 35127 Padova, Italy; filippo.agresti@cnr.it (F.A.); luca.pezzato@cnr.it (L.P.); 3 Gran Sasso National Laboratory (LNGS), National Institute of Nuclear Physics (INFN), Assergi, 67100 L’Aquila, Italy; bucci@lngs.infn.it (C.B.); daniele.cortis@lngs.infn.it (D.C.); serena.deramo@lngs.infn.it (S.D.); shihong.fu@lngs.infn.it (S.F.); cristina.giancarli@lngs.infn.it (C.G.); antonio.daddabbo@lngs.infn.it (A.D.); 4 Department of Industrial and Information Engineering and Economics (DIIIE), Università degli Studi dell’Aquila, Monteluco di Roio, 67100 L’Aquila, Italy

**Keywords:** ultra-pure aluminum, dynamic elastic modulus, cryogenic applications, microstructural characterization, crystallographic texture

## Abstract

In fundamental physics, sensors operating below liquid helium temperatures are highly vulnerable to vibrations, which can affect the sensitivity, for example, of high-performance particle detectors. Pulse-tube refrigerators, while generating vibrations lower than those of conventional systems, may still introduce several disturbances. Hence, flexible thermal connections are a commonly used mechanical solution to mitigate these undesirable effects. Among the materials that can be used, ultra-high-purity aluminum (UHP-Al) has attracted the attention for low-amplitude-vibration cryogenic applications, including gravitational wave interferometry, quantum information systems, precision space instrumentation, and cryogenic resonators. Thus, the aim of the paper is the characterization of the mechanical and microstructure properties of three UHP-Als (i.e., 5N—99.999 wt%, 5N5—99.9995 wt% and 6N—99.9999 wt%) intended for the production of thermal flexible connections with low stiffness, specifically designed to reduce vibration transmission in cryogenic environments. Mechanical properties were evaluated through standard tensile tests from room (+25 °C) to low temperature (i.e., −150 °C), providing insights into yield strength, ultimate tensile strength, elongation and elastic modulus. In addition, the dynamic elastic modulus of material loads, at cryogenic conditions (i.e., about −180 °C), was determined by measuring the natural resonance frequency, thereby assessing the material’s response to vibrational. Moreover, an extensive microstructural analysis was conducted using electron backscatter diffraction and x-ray diffraction. The correlation between the observed microstructure and the elastic properties was systematically examined. The results underscore the pivotal role of microstructural characteristics in dictating the elastic behavior of UHP Als. Eventually, the analysis provides valuable guidelines for the materials employment inside cryogenic systems, where severe vibration control is critical to maintain high operational performance.

## 1. Introduction

In fundamental physics, sensors operating below liquid helium (LHe) temperatures are highly vulnerable to vibrations, which can affect the sensitivity, for example, of high-performance particle detectors. Pulse-tube refrigerators (PTR), while generating vibrations lower than those of conventional systems, may still introduce several disturbances. Hence, flexible thermal connections are a commonly used mechanical solution to mitigate these undesirable effects [1]. Among the materials that can be used, ultra-high-purity aluminum (UHP-Al) has attracted the attention for low-amplitude-vibration cryogenic applications, including gravitational wave interferometry, quantum information systems, precision space instrumentation, and cryogenic resonators. In these systems, mechanical stability and low internal damping are essential, as even minute sources of mechanical loss can severely compromise measurement sensitivity and device performance [2,3,4].

Aluminum’s high thermal conductivity, low density, and excellent ductility make it a compelling candidate for cryogenic structural components such as flexible thermal connections, and these advantages are further amplified when impurity levels are reduced to the ultra-pure regime (≥99.999 wt.% Al) [5]. Whereas the thermal conductivity was already studied in a recent publication of the same research team [6] this work aims to focus on the mechanical properties of ultra-pure aluminum grades at cryogenic temperatures and to correlate them with microstructural features.

The microstructure and, consequently, the mechanical properties of ultra-pure aluminum are fundamentally distinct from those of commercial purity and conventional alloy grades due to the near absence of solute atoms, intermetallic particles, and dispersoids, which strongly influence defect structures and deformation mechanisms [7]. In ultra-pure aluminum, microstructures typically consist of large, equiaxed grains developed through extensive recovery and recrystallization. Grain sizes may span from several hundred micrometers to multiple millimeters depending on thermomechanical history and processing route [8]. The low concentration of second-phase particles and impurities in ultra-pure aluminum reduces grain boundary pinning, enabling abnormal grain growth and large variability in texture [9,10].

Intrinsic defect populations, such as dislocations and vacancies, are profoundly affected by ultra-high purity. With minimal solute drag, dislocation densities after annealing are low, and dislocation structures tend to reorganize into low-energy configurations or annihilate through recovery processes [11]. At cryogenic temperatures, the lack of solute interactions enhances dislocation mobility and leads to high strain hardening rates, as recently demonstrated in tensile deformation studies showing increased dislocation density and unique strain hardening behavior at −196 °C [8].

Crystallographic texture evolution is another key microstructural feature influencing mechanical behavior and elastic anisotropy. Thermomechanical processing such as rolling, extrusion, or severe deformation introduces preferred orientations, and subsequent annealing or recovery may promote texture modification or abnormal grain growth [12,13].

In this context, the knowledge of the elastic behavior of a material (i.e., elastic modulus) is fundamental to predicting the vibration response of a system or component. The elastic modulus (E) evaluation can be done through different experimental methods, such as standard tensile tests or dynamic methods (i.e., resonance frequencies analysis). Under some circumstances, the last one can be preferred to reduce the influence of mechanical uncertainties of standard tensile tests, like miss alignments of the specimens, or sliding of the gripping tools [DC5.1]. In fact, the measurement accuracy in the determination of E cannot be neglected. For example, in some polycrystalline materials, E can exhibit significant anisotropy due to crystallographic texture, the preferential orientation of grains and thermal processing [14,15].

Even if the impurities in UHP-Als are extremely low, some trace undesired elements and residual contaminations at ppm levels can affect the interactions between defects and the microstructure. These elements may segregate at grain boundaries or interact with dislocations, modifying the grain boundary mobility and dislocation damping [16]. The behavior of commercial pure Al (i.e., AA1060) under cryogenics conditions has been the subject of some recent investigations but no works related UHP-Als are currently available in the literature. For example, the AA1060 significantly increases both tensile strength and ductility at cryogenic temperature (−196 °C), showing a more uniform grain deformation and higher dislocations density. This evidence can be attributed to a dual-enhancement effect: the high strain hardening rate and the reduced dynamic recovery [17], demonstrating a strong correlation between microstructure features and mechanical performance.

Thus, the aim of the paper is the characterization of the mechanical and micro-structure properties of three UHP-Als (i.e., 5N—99.999 wt%, 5N5—99.9995 wt% and 6N—99.9999 wt%) and the investigation of the effects of the purity level on dynamic vibration response, focusing on low and cryogenic temperatures. Mechanical properties were evaluated through standard tensile tests from room (+25 °C) to low temperature (i.e., −150 °C), providing insights into yield strength, ultimate tensile strength, elongation and elastic modulus. In addition, the dynamic elastic modulus of material loads, at cryogenic conditions (i.e., about −180 °C), was determined by measuring the natural resonance frequency, thereby assessing the material’s response to vibrational. Moreover, an extensive microstructural analysis was conducted using electron backscatter diffraction and x-ray diffraction. The correlation between the observed microstructure and the elastic properties was systematically examined. The results underscore the pivotal role of microstructural characteristics in dictating the elastic behavior of UHP Als. Eventually, the analysis provides valuable guidelines for the material’s employment inside cryogenic systems, where severe vibration control is critical to maintain high operational performance.

## 2. Materials and Methods

The aim of this work is to study the mechanical and microstructural characterization of three different pure aluminums, for which composition is reported below, provided in plate form with different thicknesses (Laurand Associates Inc, Boca Raton, FL, USA):

- 5N—99.999 wt% Al (12 mm)
- 5N5—99.9995 wt% Al (12 mm)
- 6N—99.9999 wt% Al (6.5 mm)

**Figure 1:**
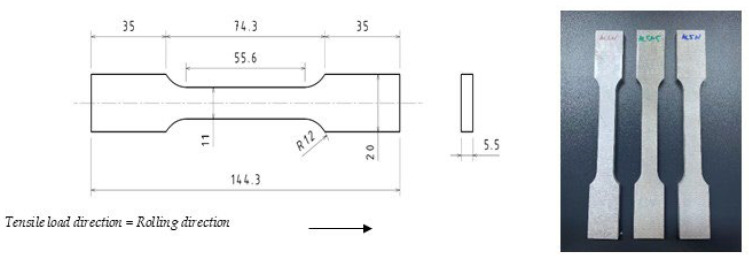
The left flat cross-section of specimens’ geometry according to EN ISO 6892-1/3 (dimensions are expressed in millimeters) and the right flat cross-section of specimens after the electrical discharge machine cutting.

Standard tensile tests were performed on flat cross-section specimens design according to the EN ISO 6892-1/3, Annex A/D [18], Figure 1. Specimens were extracted from different high-purity aluminum plates by means of no-contact cutting electrical discharge machine, keeping the specimen’s longitudinal axis (i.e., tensile load direction) parallel to the plates rolling direction (Figure 1 on the right).

Tests were conducted at room and low temperatures using a universal testing machine (i.e., INSTRON 68F100M3710, Instron^®^, Norwood, MA, USA) equipped with liquid nitrogen (LN_2_) and a climatic chamber (INSTRON 3119-610, Instron^®^, USA) able to reach −150 °C. Data were acquired using a constant strain rate of 0.00025 ± 20% s^−1^ for the determination of yield strength (YS), ultimate tensile strength (UTS), elongation (A), and 0.00007 ± 20% s^−1^ for the evaluation of the elastic modulus (E_s_) under elastic regime with multiples loading-unloading cycles. For this purpose, two kinds of extensometer have been employed: (i) standard axial clip-on extensometer (i.e., INSTRON 2630-105, Instron^®^, USA) to record the full elongation at break of the specimen up to −150 °C and (ii) a biaxial clip-on extensometer (i.e., INSTRON 2650-561, Instron^®^, USA), with of three independent channels, two for the axial and one for the transversal strains, to evaluate the E_s_ with higher accuracy up to −100 °C. The estimation of uncertainty was done, according to the Annex K of the above EN ISO 6892-1 standard, by repeated measurements considering a 95% confidence level.

**Figure 2:**
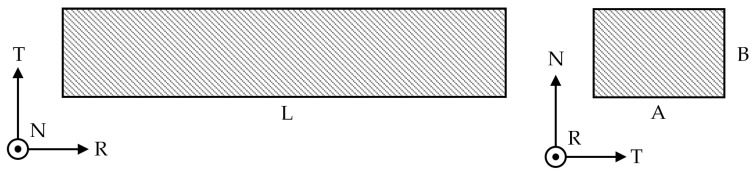
Dimensions and orientations of the samples for the measurement of the dynamic elastic modulus.

The experimental evaluation of the dynamic elastic modulus (E_d_) was done by resonance frequency measurements using prismatic samples with rectangular cross-section (Table 1 and Figure 2).

**Figure 3:**
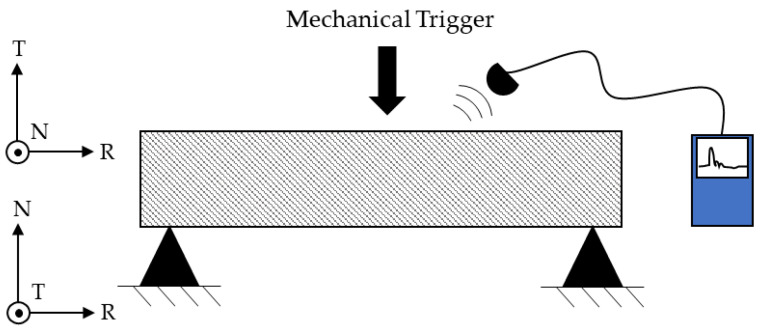
Resonance frequency measurement test with the two orientation configurations.

Dynamic modulus values were obtained by recording the sample’s flexural resonance frequency, following ASTM E1876-22 standards [19]. The samples were set into vibration by applying a mechanical impulse at the center, with the setup configured as a beam resting on two supports Figure 3. Measurements were taken by positioning the sample first on the RT plane, then rotating it 90° around the R axis to position it on the RN plane. The acoustic signal was recorded using different types of microphones. The time-domain data were converted into the frequency domain using dedicated software based on fast Fourier transform (FFT) analysis. Measurements were repeated with different acquisition devices and commercial software, showing no significant differences in the frequency spectra. The reported results represent the average of at least five measurements for each configuration, thereby validating the measurement methodology. Using the first fundamental resonant frequency of these two configurations, the dynamic elastic modulus of the material was calculated. The specimens were evaluated at cryogenic temperatures, with the resonance frequency measured immediately after withdrawal from the liquid nitrogen and maintained within the cool nitrogen vapor atmosphere built-up over the LN2 bath. Using a thermocouple welded to the specimen, the temperature was estimated to be approximately −180 °C. Tests repeated at different time intervals after removal of the specimen from the liquid nitrogen bath confirmed the stability of the resonance frequency over an extended period and across a wide temperature range, thereby ensuring good repeatability of the measurements even under non-fully steady-state conditions.

For the microstructural analyses, samples were taken from the sheets and prepared using standard metallographic techniques, ending with electropolishing in Struers A2 electrolyte at 20 V for 60 s. A surface completely free from residual mechanical deformations is essential for observing the microstructure under a scanning electron microscope (LaB_6_—EVO MA10, Zeiss^®^, Oberkochen, Germany), where channeling contrast with backscattered electrons is used as the imaging method. These prepared samples were also analyzed by XRD. Data were obtained on a Malvern Panalytical^®^ Empyrean (Malvern Panalytical, Monza Brianza, Italy) diffractometer, with CuKα radiation operating at 40 kV and 40 mA, using the Schulz geometry. Pole figures have been built tracking the scattering intensities of several reflections for different combinations of the azimuthal sample angle ø (0–360°) and the tilt angle χ (0–75 °) with steps of 5° and counting times of 10 s. X’Pert Texture software 1.2 (Panalytical) was used to elaborate the texture data with proper defocusing correction. The ODF (orientation distribution function) has been calculated from experimental pole figures and reconstructed pole figures of the diffraction planes (111) and (200) have been calculated consequently.

In order to investigate the crystallographic orientation and grains deformation features of the specimens an EBSD analysis was carried out on the RT section with a TESCAN Solaris FE-SEM (Assing S.p.a., Milano, Italy) at 5 nA, 25 kV and with a scan step of 0.3 µm, using Oxford Instruments AZtecCrystal 4.0 software for image post processing. All microstructural analysis was performed on the samples at room temperature before any cryogenic treatment.

## 3. Results

This section reports the experimental results obtained from the mechanical and microstructural characterization of the 5N, 5N5 and 6N aluminum sheets. The results are presented in separate subsections, covering tensile behavior at room and cryogenic temperatures, elastic modulus measurements, and microstructural and crystallographic analyses.

### 3.1. Tensile Tests

**Figure 4:**
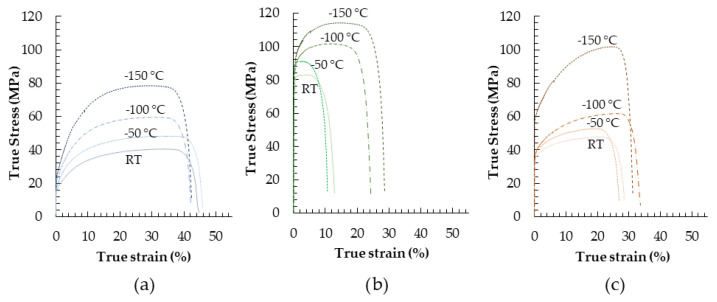
Standard tensile tests at different temperatures: (**a**) 6N—99.9999 wt%, (**b**) 5N5—99.9995 wt% and (**c**) 5N—99.999 wt%. (Engineering curves).

The results of standard tensile tests show a significant difference in the mechanical behavior of the three UHP-Als, as illustrated by the stress–strain plots in Figure 4. At room temperature (+25 °C), the 6N exhibits the lowest strength (i.e., YS and UTS) but higher ductility with respect to 5N and 5N5. This behavior is related to the greater strain-hardening capability, reflected by a significant difference between the YS and the UTS. On the other hand, the 5N5 is characterized by a limited strain-hardening. As a consequence, the plastic deformation is rapidly localized, leading to an early formation of the necking at relatively low plastic strain. Despite the limited ductility, the 5N5 exhibits the highest strength among the materials investigated. Finally, the 5N shows intermediate behavior, maintaining good ductility. Considering the small differences in chemical composition between the three materials, the observed variations in mechanical properties cannot be attributed only to the purity levels but also to the microstructure, which will be addressed and discussed in the following paragraphs. At lower temperatures, there was a significant increase in the UTS, together with a less pronounced increase in the YS for all materials, showing an enhanced strain-hardening capability. Regarding the ductility, for 6N there is no appreciable sensitivity to temperature, showing an A% nearly constant over the entire investigated temperature range (i.e., about 40%). On the contrary, for the 5N a slight increase in A% is observed with decreasing temperature. Eventually, a completely different behavior is exhibited by 5N5, which shows a substantial increase in ductility as temperature decreases. In particular, the A% increases approximately 100%, indicating a significant delay in the appearance of strain localization and necking. This effect becomes highly evident at temperatures below −100 °C, where plastic instability is strongly postponed. The experimental data are summarized in Table 2. Data without uncertainty refers to test conditions carried out only once time due to the few specimens available.

For all samples, decreasing the testing temperature leads to a significant increase in ultimate tensile strength, accompanied by a less pronounced increase in yield strength. As a consequence, a clear increase in the ultimate tensile strength to yield strength ratio is observed, indicating an enhanced strain-hardening capability at lower temperatures.

Regarding ductility, the 6N alloy shows no appreciable sensitivity to temperature, with the elongation to failure remaining nearly constant over the entire investigated temperature range. In the case of the 5N alloy, a slight increase in elongation to failure is observed with decreasing temperature.

A markedly different behavior is exhibited by the 5N5 alloy, which shows a substantial increase in ductility as temperature decreases. In particular, the elongation to failure increases by approximately 100%, indicating a significant delay in the onset of strain localization and necking. This effect becomes especially pronounced at temperatures below −100 °C, where plastic instability is strongly postponed. The quantitative results are reported in Table 2, where the mean values and associated experimental errors are provided, whenever available, for the tests performed under the specified conditions.

### 3.2. Elastic Modulus Measuraments

**Figure 5:**
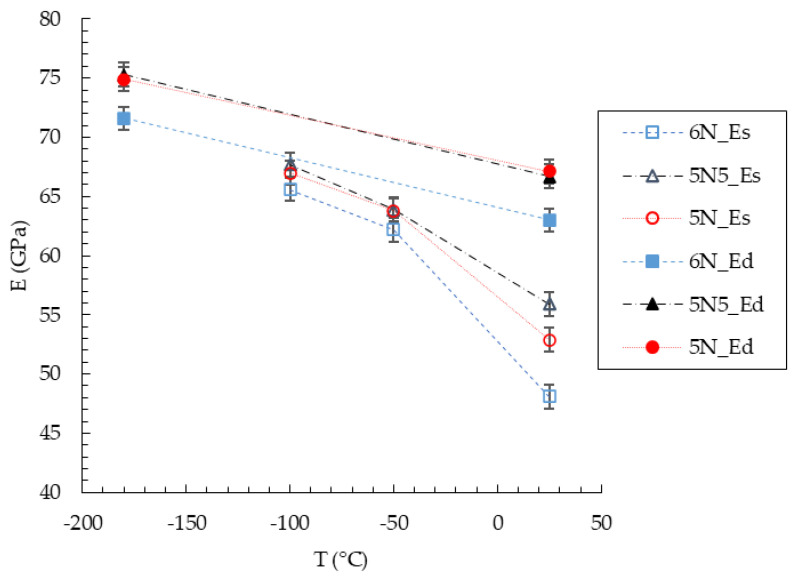
Effect of the temperature on the elastic modulus measured by tensile test (empty symbols) and by vibrational method (full symbols).

Calculated values of static elastic moduli (E_S_), evaluated from the tensile test, for the different ultra-pure aluminum at different temperatures are reported again for sake of comparison in Table 3 and Figure 5.

The dynamic elastic modulus (E_d_) was obtained using the standard ASTM E1876-22 [19] with the equation:

(1) $$E_{d}=0.9465\left(\frac{mf_{f}^{2}}{b}\right)\left(\frac{L^{3}}{t^{3}}\right)T_{1}$$

This equation permits to calculate the dynamic Young’s modulus (E_d_). *f_f_* is the fundamental resonant frequency [Hz], m the mass [g], and b, t and L are the width, the thickness and the length of the bar [mm], respectively. In particular, b is the width of the rectangular cross-section of the bar (measured along the lateral dimension perpendicular to the vibration direction), t is the thickness (measured along the dimension parallel to the direction of flexural vibration), and L is the length of the specimen measured along its longitudinal axis. Finally, T_1_ is a geometrical correction factor that takes into account the Poisson’s ratio, considered equal to 0.33 in all the cases. The results, also for the dynamic elastic moduli, are listed and displayed in Table 3 and Figure 5.

The elastic modulus clearly shows an increase with decreasing temperature for all investigated materials. This trend is consistent with the reduction in thermal vibrations and the associated stiffening of interatomic bonds, confirming the significant influence of temperature on the elastic response of ultra-high-purity aluminum over the investigated temperature range. Although both static and dynamic techniques show the same qualitative temperature dependence, a clear and systematic discrepancy between the two measurement methods is observed. Under identical temperature and loading direction conditions, the static elastic modulus obtained from tensile tests is consistently lower than the corresponding dynamic modulus. This difference highlights an intrinsic limitation of tensile-based modulus determination when applied to ultra-high-purity aluminum, particularly in the presence of very low yield stresses.

During tensile testing, the strain amplitudes required to evaluate Young’s modulus are not negligible compared to the elastic limit of the materials. As a result, time-dependent, viscous, or incipient plastic deformation mechanisms may be activated even at nominally elastic strains, leading to a systematic underestimation of the elastic modulus. This effect is further enhanced by the relatively low strain rates employed in the tensile tests, which can promote microplastic deformation and reduce the apparent stiffness. In contrast, resonance frequency measurements involve extremely small strain amplitudes that remain well within the purely elastic regime, providing a more intrinsic measure of the elastic response. Both techniques also reveal measurable differences in elastic modulus among the three ultra-pure aluminums, particularly between the 6N and the 5N and 5N5. These differences are more pronounced at room temperature and tend to decrease with decreasing temperature; however, in the case of dynamic measurements, significant differences persist even under cryogenic conditions. Given the negligible compositional differences among the materials, these variations cannot be attributed to chemical effects and instead reflect the influence of crystallographic and microstructural conditions, that are considered in next sections of this work.

### 3.3. Microstructural Charactherization

SEM analyses were conducted on the electropolished cross-sections of the tensile samples (NT plane). Using backscattered electrons, crystallographic contrast was achieved, aided by partial metallographic etching from the electropolishing process, which allowed observation of the specific substructure characterizing each sample.

**Figure 6:**
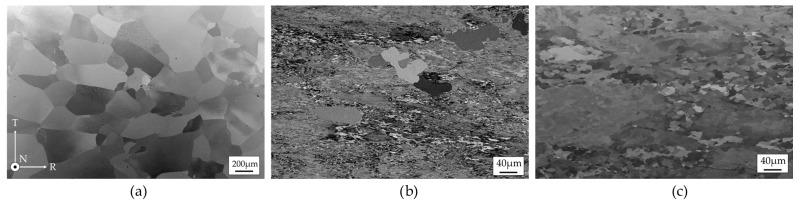
Back-scattered electron images after electropolishing. (**a**) 6N, (**b**) 5N5 and (**c**) 5N. On the corner on the left, the reference of the directions. Note that scale of (**a**) is different from the ones of (**b**) and (**c**) to properly identify the different microstructural features.

In particular, sample 6N, as confirmed by its mechanical properties, exhibits a fully recrystallized equiaxed structure, with grains free from defects or substructures associated with deformation processes (Figure 6a).

In the case of the 5N5 sample (Figure 6b), the highest strength is attributed to a highly fragmented substructure resulting from deformation. A notable feature of this sample is the presence of large, recrystallized grains beginning to nucleate and grow, consuming the deformed matrix (a sign of possible heat treatment either concurrent with or following deformation). The microstructure of sample 5N (Figure 6c) suggests that, following or during the deformation, the material underwent a heat treatment that partially restored its mechanical properties, through a recovery process that contributed to the formation of a substructure with a reduced number of dislocations.

### 3.4. EBSD Analysis

**Figure 7:**
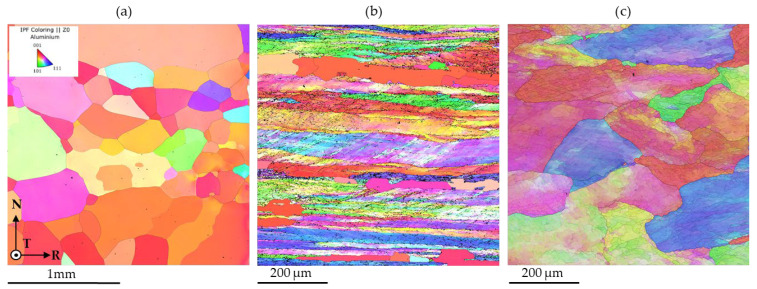
Inverse pole figure (IPF) maps of the samples (**a**) 6N, (**b**) 5N5 and (**c**) 5N. Note that scale of (**a**) is different from the ones of (**b**) and (**c**) to properly identify the different microstructural features.

The EBSD analyses further confirm the trends previously identified through mechanical testing and SEM characterization. The 6N exhibits a very coarse-grained microstructure, characterized by predominantly equiaxed grains with no evident signs of plastic deformation. The inverse pole figure (IPF) maps (Figure 7a) indicate that most grains are oriented close to the ⟨001⟩ direction with respect to the normal direction of the analyzed plane, in agreement with the strong CUBE texture identified by X-ray diffraction, reported in the next chapter. The microstructure is dominated by high-angle grain boundaries as reported in Figure 7a, consistent with a fully recrystallized condition and a low dislocation density.

**Figure 8:**
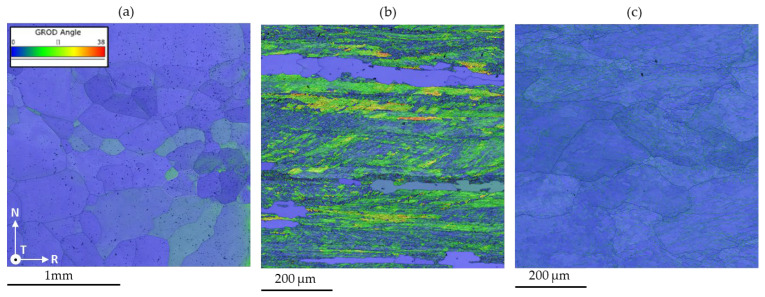
Grain orientation deviation (GROD) maps of samples (**a**) 6N, (**b**) 5N5 and (**c**) 5N. Note that scale of (**a**) is different from the ones of (**b**) and (**c**) to properly identify the different microstructural features.

In contrast, the EBSD results for 5N reveal a highly deformed microstructure. Figure 7b shows elongated grains aligned along the rolling direction, indicating a strong deformation-induced anisotropy. A high density of dislocation substructures is evident, with the formation of numerous low-angle grain boundaries associated with intense lattice distortion. This condition is further supported by the grain orientation deviation (GROD) maps (Figure 8b), which display high GROD values, indicative of significant intragranular misorientation and a high dislocation density. In addition, localized regions exhibiting low GROD values are observed, which can be associated with recrystallized grains, suggesting the occurrence of partial recrystallization as a result of prior deformation and possible thermal exposure.

The 5N alloy presents a recovered microstructural condition. The GROD maps reveal a generally lower lattice distortion compared to the 5N5, with GROD peaks mainly localized along cell or sub-grain boundaries within the grains, as illustrated in Figure 8c. The grains appear slightly elongated along the rolling direction, and the presence of sub-grains with relatively low dislocation content suggests a recovered microstructure. This condition is consistent with a recovery treatment, during which dislocation annihilation and rearrangement occur, leading to the accumulation of dislocations at cell boundaries while maintaining comparatively low dislocation densities within sub-grain interiors.

These microstructural features provide a consistent explanation for the mechanical behavior observed in tensile testing. The fully recrystallized, coarse-grained microstructure of the 6N accounts for its low strength and high ductility. Conversely, the heavily deformed and dislocation-rich microstructure of the 5N5 results in high strength due to dislocation strengthening, albeit at the expense of reduced dislocation mobility and ductility. The 5N exhibits a balanced combination of strength and deformability, arising from its microstructure enriched with dislocation substructures that enhance strength, while the low-dislocation-density regions within sub-grains allows for a moderate degree of plastic deformation.

### 3.5. XRD and Pole Figures

**Figure 9:**
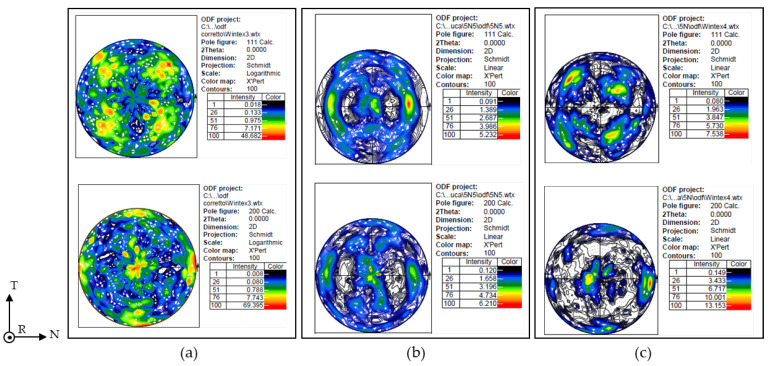
Reconstructed pole figure along the direction <111> and <200> of samples (**a**) 6N, (**b**) 5N5 and (**c**) 5N. The directions on the left indicate the geometrical reference of the pole figures.

**Figure 10:**
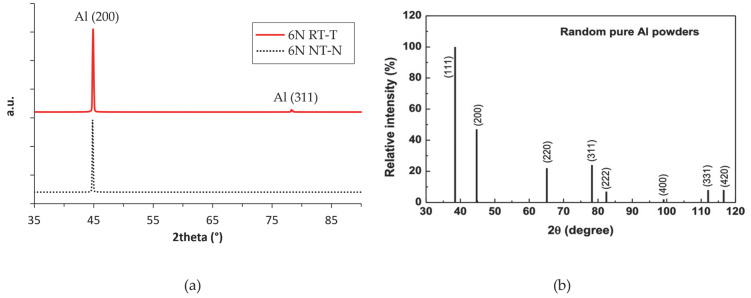
(**a**) Sample after electropolishing, XRD pattern of sample 6N with respect to the directions RT—T and NT—N. (**b**) XRD peak intensities and positions for a reference randomly oriented pure Al [20].

Deformation and recrystallization processes introduce textures and preferred orientations of crystalline domains in the sheets from which specimens for mechanical tests were extracted, leading to material properties’ anisotropies. The microstructural analysis is in agreement with the mechanical properties obtained during the tensile tests. The primary objective of this study is to determine the elastic moduli of three different ultra-pure aluminums. This parameter is intrinsically linked to the crystalline structure of the material and, consequently, its orientation. Specifically, the potential presence of textures in the analyzed samples was investigated, with particular focus on the 6N sample, which exhibited lower stiffness according to dynamic elastic modulus measurements. Preliminary XRD analysis of the 6N sample in Figure 9a revealed a strong preferred orientation. By analyzing both the RT and NT surfaces, a pronounced peak for planes parallel to the (200) plane, indicating an alignment of the unit cell with the geometries of the samples used for mechanical characterization, has been observed. As a reference, Figure 10b shows the peak intensities of randomly oriented aluminum powder.

To further deepen the analysis, additional investigations were carried out on the crystallographic orientation of the investigated materials by means of X-ray diffraction texture measurements. Texture analyses were performed through the evaluation of the {111} and {200} pole figures, which provide key information on the preferred crystallographic orientations developed during processing (Figure 9).

The 6N alloy, analyzed on the section perpendicular to the rolling direction (R), corresponding to the transverse (T) and normal (N) planes, exhibits a very strong CUBE-type texture. The intensity of this texture is extremely pronounced, to the extent that a logarithmic scale was required in order to properly visualize and quantify the pole figure maxima, and also because of the coarse grain size. The {111} and {200} pole figures show well-defined and highly concentrated intensity peaks, indicating a strong preferential alignment of ⟨100⟩ directions with respect to the sample reference frame.

The 5N alloy also shows a tendency toward a cube-type texture; however, the intensity of this component is significantly lower compared to the 6N material. As a result, the corresponding pole figures could be represented using a linear intensity scale. The maxima appear broader and less intense, suggesting a weaker and more dispersed crystallographic alignment. In contrast, the 5N5 alloy displays a markedly different texture, characteristic of rolled aluminum materials. The {111} and {200} pole figures reveal a distribution of intensity consistent with typical rolling texture components, with no dominant cube orientation. The intensity is more evenly spread across the pole figures, indicating a more complex and heterogeneous orientation distribution. Overall, the pole figure analyses clearly highlight substantial differences in crystallographic texture among the three ultra-high-purity aluminums, which will be further discussed in the following sections in relation to the mechanical behavior.

## 4. Discussion

The tensile behavior observed at room temperature (Figure 4) highlights a clear correlation between mechanical properties and the microstructural condition of the investigated ultra-high-purity aluminum. Despite the extremely small differences in chemical composition among the 6N, 5N, and 5N5 alloys, pronounced variations in strength, ductility, and strain-hardening behavior are evident as evidenced in Table 2, confirming that compositional effects play a negligible role. Instead, these differences are primarily governed by microstructural features such as grain size, dislocation density, substructure development, and crystallographic texture, in agreement with established strengthening theories for high-purity FCC metals [21].

The 6N alloy exhibits the lowest yield strength and ultimate tensile strength, coupled with the highest elongation to failure (Table 2). This mechanical response is characteristic of a fully recrystallized, coarse-grained microstructure as evidenced by SEM and EBSD analyses (Figure 6a and Figure 7a). The predominance of high-angle grain boundaries Figure 8a) and the absence of deformation-induced substructures facilitate dislocation glide and multiplication during plastic deformation, resulting in a pronounced strain-hardening capability. The large ratio between ultimate tensile strength and yield strength delays the onset of plastic instability and necking, thereby promoting uniform deformation and high ductility, consistent with classical descriptions of strain hardening in high-purity aluminum [22].

In contrast, the 5N5 alloy displays the highest strength but the lowest ductility at room temperature (Table 2). The EBSD results reveal a highly deformed microstructure characterized by elongated grains, a high density of low-angle grain boundaries, and significant intragranular misorientation (Figure 7b and Figure 8b). These features indicate intense lattice distortion and a high dislocation density. However, the limited capacity for additional dislocation storage during deformation leads to a reduced strain-hardening rate, as evidenced by the ultimate tensile strength to yield strength ratio approaching unity. As a result, plastic deformation rapidly localizes, promoting early necking and limited uniform elongation, a behavior well documented in heavily cold-worked aluminum [23].

The 5N alloy exhibits intermediate mechanical behavior, which can be directly related to its recovered microstructure. The presence of subgrains with relatively low dislocation densities in their interiors, combined with dislocation accumulation at subgrain boundaries (Figure 6c, Figure 7c and Figure 8c), provides a balance between strength and deformability. Such recovered microstructures are known to enhance yield strength while retaining sufficient dislocation mobility to sustain moderate strain hardening [24].

### 4.1. Effect of Temperature on Mechanical Properties

The temperature-dependent tensile results reveal a consistent increase in ultimate tensile strength with decreasing temperature for all investigated materials, accompanied by a less pronounced increase in yield strength (Table 2). This leads to a systematic increase in the ultimate tensile strength to yield strength ratio, indicating enhanced strain-hardening capability at lower temperatures. This behavior, typical of FCC materials, is commonly attributed to the suppression of thermally activated dislocation recovery mechanisms and reduced cross-slip, which promotes greater dislocation accumulation during plastic deformation [25,26].

The influence of temperature on ductility strongly depends on the initial microstructural state. The 6N alloy shows negligible sensitivity to temperature in terms of elongation to failure, indicating that its fully recrystallized microstructure already provides optimal conditions for homogeneous plastic deformation.

The 5N alloy exhibits a slight increase in elongation to failure with decreasing temperature. This trend can be attributed to enhanced strain hardening that delays strain localization.

A markedly different behavior is observed for the 5N5 alloy, which shows a substantial increase in ductility as temperature decreases, with elongation to failure approximately doubling at cryogenic temperatures. This pronounced improvement indicates a significant delay in the onset of plastic instability and necking, particularly below −100 °C. At low temperatures, the enhanced strain-hardening capability compensates for the initially limited hardening at room temperature, allowing the dislocation-rich microstructure to accommodate larger uniform strains before localization, as also observed in other FCC metals like Cu or austenitic stainless steels [27,28].

### 4.2. Comparison Between Static Versus Dynamic Elastic Modulus Evaluation

Both static and dynamic measurements show a clear increase in elastic modulus with decreasing temperature for all the considered ultra-pure aluminums (see, Figure 5 and Table 3), consistent with reduced thermal vibrations and the stiffening of interatomic bonds. This temperature dependence of Young’s modulus is well established for aluminum and other FCC metals [29].

However, a systematic discrepancy between static elastic moduli derived from tensile tests and dynamic moduli obtained via resonance frequency measurements is consistently observed, with static values being lower (Figure 5 and Table 3). This difference highlights the intrinsic limitations of tensile-based modulus determination for ultra-high-purity aluminum, where extremely low yield stresses make the separation between purely elastic and microplastic deformation difficult [28].

During tensile testing, the strain amplitudes used to determine Young’s modulus are not negligible relative to the elastic limit. As a result, incipient plasticity, time-dependent anelastic effects, or microplastic deformation may occur even within the nominally elastic regime, leading to an apparent reduction in stiffness. These effects are further amplified by the low strain rates typically employed in tensile testing. In contrast, resonance frequency measurements involve extremely small strain amplitudes that remain well within the linear elastic regime, providing a more intrinsic measure of Young’s modulus.

Despite these methodological differences, both techniques reveal measurable differences in elastic modulus among the three ultra-pure aluminums.

### 4.3. Role of Texture and Crystallographic Orientation

Texture analysis provides additional insight into the observed mechanical and elastic behavior. The 6N alloy (Figure 9a) exhibits an exceptionally strong cube-type texture, typical of fully recrystallized aluminum, which favors easy slip activation along ⟨100⟩ directions. This texture contributes to low yield strength, high ductility, and anisotropic elastic behavior

The 5N alloy (Figure 9c) retains a weaker cube-type texture, consistent with partial recovery rather than complete recrystallization. The broader pole figure maxima indicate a more dispersed orientation distribution, In accordance with its intermediate strength and ductility [19,30].

In contrast, the 5N5 alloy Figure 9b exhibits a typical rolling texture, with no dominant cube component. Such deformation textures are known to increase strength through orientation-dependent slip resistance while reducing ductility due to limited compatibility between neighboring grains [31,32,33,34].

Overall, these results demonstrate that in ultra-high-purity aluminum, mechanical strength, ductility, strain-hardening behavior, and elastic response are overwhelmingly governed by microstructural and crystallographic conditions rather than chemical composition. The obtained values of mechanical properties also at cryogenic temperatures demonstrate the possibility to employ ultra-pure aluminum for applications in extreme low-temperature conditions.

## 5. Conclusions

This study shows that ultra-high-purity aluminum is well suited for low-vibration cryogenic applications, provided that its microstructural state and texture are properly controlled. The mechanical and elastic properties are only weakly dependent on nominal purity, while they are strongly affected by processing history and crystallographic orientation.

All investigated ultra-pure aluminums exhibited a clear increase in elastic modulus with decreasing temperature, indicating a progressive stiffening of interatomic bonds under cryogenic conditions. This effect is particularly relevant for the design of flexible thermal links, where stiffness directly influences vibration transmission. Dynamic resonance measurements proved essential to reliably detect this temperature dependence, as static tensile tests tend to underestimate the elastic modulus in ultra-pure aluminum due to incipient microplasticity.

Regarding deformation behavior, the results show that the effect of temperature on ductility in ultra-pure aluminum is strongly influenced by the initial work-hardening state and texture. In particular, increasing the degree of work hardening makes the recovery of deformability at low temperatures more pronounced, as observed in the heavily deformed 5N5. At cryogenic temperatures, enhanced strain hardening delays plastic instability and promotes a significant increase in elongation to failure, partially compensating for the limited ductility observed at room temperature.

Overall, these results indicate that by tailoring thermomechanical processing and texture, ultra-pure aluminum components can be optimized to achieve low stiffness at room temperature, controlled stiffening at cryogenic temperatures, and reliable mechanical performance in vibration-sensitive cryogenic systems.

## Figures and Tables

**Table 1:** Mass and size of the samples prepared for the calculation of the dynamic Young’s modulus.

	5N	5N5	6N
**Mass [g]**	13.5	13.208	14.735
**Length L [mm]**	76.33	68.71	70.75
**Prismatic section B [mm]**	6.02	6.26	6.53
**Prismatic section A [mm]**	11.92	11.45	11.92

**Table 2:** Standard tensile test results at different temperatures: YS, UTS, A% and E_s_.

	Temperature (°C)	6N	5N5	5N
**YS** **(MPa)**	+25	16.4 ± 1.3	74.6 ± 3.7	34.1 ± 1.1
	−50	19.0 ± 2.3	82.9 ± 1.6	35.5
	−100	21.2	88.5 ± 3.8	37.0
	−150	24.2	83.1	58.5
**UTS** **(MPa)**	+25	40.4 ± 0.3	82.9 ± 0.6	48.1 ± 1.5
	−50	48.5 ± 0.9	91.8 ± 1.9	52.7 ± 0.1
	−100	59.6 ± 0.1	101.7 ± 0.3	61.8 ± 0.1
	−150	78.5 ± 0.2	114.2 ± 0.3	101.9 ± 0.3
**A** **(%)**	+25	48.4 ± 6.3	11.6 ± 3.7	27.1 ± 4.5
	−50	44.4 ± 2.9	10.2 ± 1.9	26.9
	−100	42.2 ± 0.1	24.3	33.7
	−150	42.4	27.8	31.2
**E_s_** **(GPa)**	+25	48.1 ± 0.6	55.9 ± 0.3	59.2 ± 0.7
	−50	62.2 ± 0.7	63.9 ± 0.1	66.2 ± 1.5
	−100	65.6 ± 1.0	67.7 ± 1.2	66.0 ± 1.3

**Table 3:** Dynamic elastic modulus (E_d_) and static elastic modulus (E_s_) obtained at room (RT) and at cryogenic temperature (−50 and−100 °C).

	Temperature	6N	5N5	5N
**E_s_** **(GPa)**	25 °C	48.1 ± 0.6	55.9 ± 0.	52.9 ± 0.3
	−50 °C	62.2 ± 0.7	63.9 ± 0.1	63.8 ± 0.5
	−100 °C	65.6 ± 1.0	67.7 ± 3.2	67.0 ± 1.0
**E_d_** **(GPa)**	25 °C	63.0 ± 1.6	66.7± 0.8	67.1 ± 1.2
	LN2 *	71.6 ± 1.2	75.3 ± 0.7	74.9 ± 1.1

* LN2 is the temperature of the sample immediately after the removal from a liquid nitrogen bath.

## Data Availability

The original contributions presented in this study are included in the article. Further inquiries can be directed to the corresponding author.
